# Alternative protein-based foods must contribute to micronutrient adequacy

**DOI:** 10.1080/03036758.2024.2304196

**Published:** 2024-01-29

**Authors:** Nick W. Smith, Andrew J. Fletcher, Warren C. McNabb

**Affiliations:** a Sustainable Nutrition Initiative®, Riddet Institute, Massey University, Palmerston North, New Zealand; b Fonterra Research & Development Centre, Palmerston North, New Zealand

**Keywords:** Mathematical modelling, human nutrition, sustainability, plant-based, scenario modelling

## Abstract

Sustainable diets must consider health, economic, environmental, and social outcomes. The development and production of alternative protein foods should also make these considerations. We examined the nutritional role of these foods, with New Zealand (NZ) as a case study.

We used the DELTA Model® to assess 2020 NZ nutrient supply. We then simulated the substitution of 50% of meat supply (by mass) with various plant protein sources (soy, peas, beans, and mushroom), and observed the impact on nutrient supply.

NZ had an undersupply of calcium (38%), vitamin E (34% deficit), dietary fibre (20%), potassium (13%), and vitamin C (10%) compared to population requirements. Contrastingly, there was sufficient protein and amino acid supply for an additional 2.4 million people. Halving of meat supply resulted in decreased availability for potassium (21% deficit), zinc (17%), folate (10%), and iron (9%).

Soy proved the best nutritional replacement for meat, with reduced deficits for all undersupplied nutrients compared to 2020. Replacement with other modelled protein sources resulted in greater nutrient deficits.

The nutritional implications beyond protein must be considered when making dietary substitutions. There are clear public health reasons to fortify alternative proteins with both the nutrients lost in substitution and those already in deficit.

## Introduction

‘Alternative proteins’ is a common term in current popular media, usually referring to products such as plant-based meat and dairy alternatives, but also to emerging technologies such as precision fermentation. Currently available products are commonly marketed towards vegetarian or vegan consumers, but increasingly on health and environmental grounds to a broader audience, evidenced by increased market share and diversity of product offering globally (Andreani et al. 2023). Indeed, much evidence points to the sustainability credentials of predominantly plant-sourced diets, with animal-sourced foods primarily contributing to ensure complete nutrition (Willett et al. 2019; FAO 2023a).

However, ‘predominantly plant-sourced diets’ does not directly necessitate highly processed alternative protein products. Simulation studies have found that minimally processed plant-sourced foods would make a more positive contribution to simulated diets than the highly processed products more commonly considered under the category of alternative proteins (Reynolds et al. 2023). There is evidence for the negative dietary impacts of ultra-processed plant-based meat alternatives, particularly due to their sodium content, but composition, structure, digestion, and resulting consequences for the consumer can vary widely between products (McClements 2023; Reynolds et al. 2023). In contrast, minimally processed legumes, nuts, and seeds are widely promoted as part of a healthy diet (National Health and Medical Research Council 2013; Ministry of Health 2020a; U.S. Department of Agriculture and U.S. Department of Health and Human Services 2020). Thus, a tension exists between the value of a minimally-processed, predominantly plant-sourced diet and the consumer movement towards ultra-processed plant-based food products.

The extent to which alternative proteins should contribute to sustainable, healthy diets must be considered on a combined environmental, economic, health, and nutrition basis. Here, we contribute evidence to support the nutrition aspect of the discussion, by simulating the nutritional potential of plant foods commonly used in the production of a broad range of existing plant-based meat alternatives when applied at a national scale in New Zealand (NZ). We chose to focus on plant foods used in the production of diverse meat alternatives (as opposed to alternative proteins fitting elsewhere in the diet), due to recent scientific focus on meat substitution in NZ (Drew et al. 2020; McDowell et al. 2022; Reynolds et al. 2023) and methodological and data challenges in simulating increases in other alternative proteins. We extend the existing literature, which has largely focussed on diets for individuals or the average diet of a population, by taking a national food supply approach. The assumption is that if nutrients are undersupplied in total food supply compared to the national population’s requirements, this implies insufficient intakes within the population, thus indicating a problem to be addressed.

The aim of this study was to identify priority nutrients that would appear in shortfall in NZ nutrient supply under scenarios of meat replacement with plant foods, with the hypothesis that undersupplied nutrients would be micronutrients, in line with international evidence (Smith et al. 2021; Stevens et al. 2022). Identifying nutrient deficits will allow policy makers and alternative protein product manufacturers to make decisions on fortification and formulation of consumer products – the most obvious intervention to increase nutrient supply from these alternatives – in such a way that they promote population nutrient adequacy.

## Materials and methods

Data from the DELTA Model® (version 2.0) for NZ was extracted for this analysis. The model calculation methodology and data sources are explained fully by Smith et al. (2021), but are briefly summarised here.

The DELTA Model® takes food balance sheet data from the United Nations Food and Agriculture Organization (FAO; FAO (2023b)), which captures the total quantity of food commodities intended for human consumption in a country after consideration of trade, non-food uses, and supply chain losses. This quantity is further adjusted for consumer food waste using a second FAO source (FAO 2011).

Food commodities are then matched to food composition data published by the United States Department of Agriculture (USDA 2020), so that the total quantity of nutrients available nationally can be calculated. In the case of protein and the indispensable amino acids (IAA), values were also adjusted for digestibility using true ileal digestibility coefficients from the literature (Moughan et al. 2012). This can then be compared to national nutrient requirements, calculated using demographic data for the age and sex structure of the population and European Food Safety Authority (EFSA) and FAO nutrient reference values (NRV; (FAO 2013; European Food Safety Authority 2017)). We set target intake levels using Population Reference Intakes (PRI; where available), or either Estimated Average Requirements (EAR) or Adequate Intakes (AI) if no PRI was available. If both a PRI and an EAR were available, the EAR was displayed as the lower end of a range bar in output figures. Safe upper limits were also included in results where available. EFSA NRV and USDA compositions were used rather than NZ values as the DELTA Model® was designed for use at a global level and for international comparisons, hence the need for consistent underlying datasets.

The DELTA Model® features raised nutrient intake targets for iron and zinc for scenarios in which these nutrients are sourced largely or solely from plant foods. These are based on EFSA and World Health Organization NRV for diets with high phytate content, and equate to a 50% increase in the iron intake target and a 37% increase in the zinc intake target (World Health Organisation 2004; European Food Safety Authority 2017). These targets have been used in addition to the standard values for all simulated scenarios and are presented alongside the standard results.

The above process allows for national nutrient supply to be compared to national requirement. For simulating alternative scenarios, the food supply quantities were adjusted. Meat supply in NZ was approximately 200 g per person per day in 2020 according to DELTA Model® analysis. Therefore, a Half Meat scenario was created by halving the current meat supply. Replacement scenarios were also simulated, where 100 g per person per day of soybeans, beans, mushrooms, and peas were added to national food supply. The selection of foods to include in the replacement scenario was guided by common base ingredients for plant-based meat alternatives currently on the market.

Replacing the mass of meat (rather than the protein or energy content) was selected here as a more representative way consumers would replace meat. These partial substitution scenarios were selected rather than complete substitution as they were perceived as more realistic changes for the foreseeable future.

The nutrient compositions used for the replacement scenarios were all for raw commodities to ensure the maximum nutrient composition from the food source (before any preparation losses) was used. As per the DELTA Model®, an average of multiple compositions was used for each replacement (e.g. the average composition of white, brown, morel, chanterelle, and shiitake mushrooms was used in the mushroom scenario).

## Results

**Figure 1.**
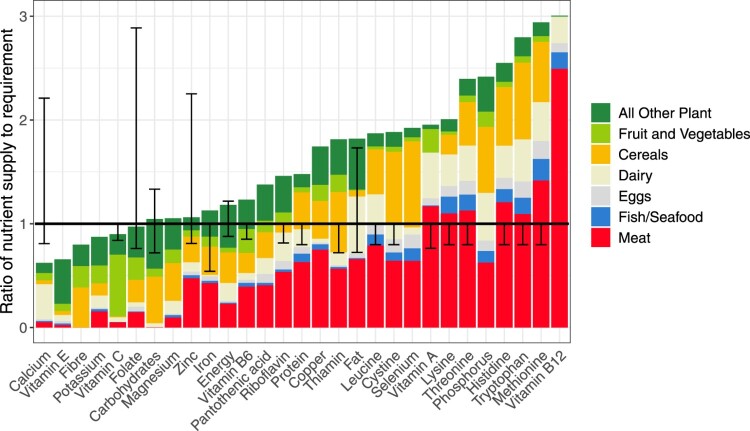
NZ national nutrient supply and food sources as a proportion of national requirement in 2020. Range bars represent safe upper intake levels (not shown when taking values greater than 3) and lower intake reference values (see methods), where available.

NZ nutrient supply compared to requirement in 2020 is shown in Figure 1 for the 29 nutrients included in the DELTA Model®.

Several nutrients, most notably calcium, vitamin E, and dietary fibre, were undersupplied compared to national requirements in 2020. Importantly, protein and the IAA were supplied at least 48% in excess of national requirements (Table 1). It was predominantly micronutrients that were undersupplied or available only marginally above national requirements.

**Table 1.** National supply of nutrients as a % of national requirement under modelled scenarios. Values 105% or below are shown in bold.

Nutrient	2020 supply	Half meat	Soy	Bean	Mushroom	Pea
Calcium	**62%**	**60%**	**79%**	**63%**	**62%**	**62%**
Vitamin E	**66%**	**65%**	**67%**	**67%**	**65%**	**66%**
Dietary fibre	**80%**	**80%**	**101%**	**91%**	**90%**	**105%**
Potassium	**87%**	**80%**	110%	**89%**	**93%**	**87%**
Vitamin C	**90%**	**87%**	106%	112%	**88%**	132%
Folate	**97%**	**90%**	169%	121%	**94%**	111%
Carbohydrates	**105%**	**105%**	110%	109%	106%	109%
Magnesium	**105%**	**101%**	146%	116%	**105%**	111%
Zinc^1^	106%	**83% (61%)**	106% (77%)	**89% (65%)**	**94% (69%)**	**95% (69%)**
Iron^1^	113%	**91% (61%)**	152% (101%)	106% (71%)	122% (81%)	**104% (69%)**
Energy	118%	107%	118%	110%	108%	111%
Vitamin B6	123%	**104%**	117%	112%	113%	115%
Pantothenic acid	138%	117%	130%	126%	145%	120%
Riboflavin	146%	119%	146%	132%	139%	128%
Protein	148%	116%	160%	128%	121%	128%
Copper	174%	137%	201%	159%	174%	152%
Thiamin	181%	153%	222%	182%	160%	186%
Fat (total lipid)	182%	149%	167%	150%	150%	150%
Leucine	187%	148%	209%	159%	151%	159%
Cystine	188%	156%	215%	166%	158%	162%
Selenium	192%	160%	171%	162%	176%	163%
Vitamin A	195%	137%	138%	138%	137%	143%
Lysine	201%	146%	212%	158%	150%	161%
Threonine	240%	183%	268%	199%	189%	202%
Phosphorus	242%	211%	277%	228%	232%	231%
Histidine	255%	195%	195%	195%	195%	195%
Tryptophan	280%	225%	330%	242%	231%	238%
Methionine	294%	223%	277%	232%	227%	239%
Cobalamin (vitamin B12)	301%	176%	176%	176%	177%	176%

^1^ Values in parentheses are the results if the intake target values for diets high in phytate are used.

Given that meat substitution was examined here, Figure 1 shows the current role of meat in NZ nutrient supply. Meat’s greatest contribution (as a proportion of current total supply) was to nutrients for which a considerable excess above national requirement existed in 2020, such as the amino acids and vitamin B12. The exceptions were zinc and iron, for which there was <15% excess above national requirement, and for which meat provided 45% and 38% of supply, respectively.

Table 1 shows total national nutrient supply as a percentage of national population requirement for 2020 and for the alternative scenarios simulated. Removing half of the current NZ meat supply resulted in increased nutrient deficits for most nutrients undersupplied in 2020, as well as resulting in new deficits for iron and zinc. Protein and the IAA were supplied at least 16% in excess of national requirement, while energy supply decreased from 18% to 7% above requirement in this scenario.

In the replacement scenarios, outcomes were highly dependent on the choice of substitute. The Soy scenario had the most positive outcome, resolving all nutrient deficits except calcium and vitamin E, and reducing the magnitude of the deficit for these nutrients compared to 2020. Even with more stringent targets, iron supply met requirements, although zinc did not. This made the Soy scenario generally an improvement on 2020 nutrient deficits.

The Bean, Mushroom, and Pea scenarios all resulted in zinc deficits of at least 5%. However, none resulted in deficits for iron using standard intake targets, although the surplus was <6% for both the Bean and Pea scenarios, compared to a 13% surplus in the 2020 data. Considering the other nutrients that were undersupplied in the 2020 data, the Pea scenario resolved the deficits for dietary fibre, vitamin C, and folate, but had a negligible impact on potassium supply. The Bean scenario increased the supply of dietary fibre, potassium, vitamin C and folate, but did not resolve dietary fibre and potassium deficits. The Mushroom scenario had the poorest results, with all 2020 nutrient deficits remaining, some larger and some smaller.

In no scenarios investigated were protein or the IAA undersupplied, noting that these results include adjustment for digestibility.

## Discussion

The DELTA Model® was used to ascertain the role of meat in current NZ nutrient supply and simulate the consequences of its partial replacement with various plant foods. The model showed that meat currently provides a notable proportion of the total national supply of many nutrients, but that this contribution is most notable for nutrients that are supplied well in excess of population requirements. The model also found that in 2020 NZ had an undersupply of calcium, vitamin E, dietary fibre, potassium, vitamin C, and folate against population requirements.

When simulating scenarios in which meat supply was halved and the same mass of various plant foods was substituted in its place, the results for nutrition were dependent on the substitute used. Substituting half of current meat supply with soy had a net positive impact on nutrient supply but did not fill all nutrient deficits. The results for other substitutes varied and were inferior to the soy scenario.

The nutrient deficits identified were largely unsurprising. Previous analysis with the DELTA Model® has identified that calcium and vitamin E are undersupplied globally, and that potassium supply only slightly exceeds global requirement (Smith et al. 2021). Dietary fibre, vitamin C, and folate have higher global supply, but undersupplies in specific countries are not uncommon. The 2008/09 NZ Adult Nutrition Survey (the most recent available) also identified high rates of inadequate calcium intake (particularly in women), whereas rates of inadequate vitamin C intake were found to be very low in most demographic groups (University of Otago and Ministry of Health 2011). Inadequate intake rates were not given for vitamin E and dietary fibre, but mean intakes were approximately equal to the EFSA recommendation in all age groups. Folate fortification of bread is now mandatory in NZ, which will not be captured in the data used in our analysis. Finally, a 2015 study with NZ participants found that only 23% had sufficient potassium intakes (McLean et al. 2015), supporting the results found here.

It is important to note the differences between national-level estimations of nutrient availability from food supply data (as performed here) and intake estimates based on nationally-representative surveys using dietary recall or food frequency questionnaire data. Beal et al. (2021) previously highlighted the differing conclusions that can be obtained depending on the approach used, due to inconsistencies between the two when inferring national intakes of specific food groups. However, each approach has advantages, with food supply data updated more frequently than intake surveys and generally more accessible and comparable through its integration into international databases. Results of the two approaches should be compared for greater insight, as performed here.

The results of this modelling should be seen as indicative rather than precise. Uncertainty exists in the underlying food composition (which is from USDA), supply, and consumer waste data. A sensitivity analysis of the model to these parameters is currently underway. Nevertheless, the directional change of nutrient supply in the investigated scenarios is instructive, highlighting likely areas of concern.

While the inclusion of 29 nutrients in the analysis is a strength of this work, it should be noted that this does not cover all nutrients essential in the diet. For example, fatty acids were not included due to their absence from the DELTA Model®, resulting from insufficient coverage of these nutrients in the underlying composition data for the food items included in the model. Bridging these gaps for a more comprehensive picture of nutrient supply is a priority for future research, but other analyses have previously shown decreased saturated fat and increased polyunsaturated fat intakes or content from similar substitution scenarios or product comparisons (Drew et al. 2020; McClements 2023), which would likely be replicated in our own scenarios.

The choice of using PRI over EAR in this research has consequences for our results. For example, vitamin C would be adequate in all scenarios using the EAR, as would iron and zinc (unless using the bioavailability adjustment for diets high in phytate). The EAR represents the average intake requirement of the population, whereas the PRI is two standard deviations greater, thus should meet the needs of almost all individuals (EFSA 2010). While the EAR is often used to estimate prevalence of inadequate intakes in a population, EFSA state that for nutrition planning for groups ‘the PRI could be a practical starting point’ for assessing nutrient adequacy, hence its adoption as the target here (EFSA 2010). We believe the use of the PRI is the most defensible as it minimises the chances of inadequate intakes given the uneven distribution of food and nutrition within a population.

A slightly surprising finding was the relatively minor changes in total energy supply between the scenarios. In the Half Meat scenario this was expected, due to the relatively low energy content of meat. However, it could have been expected that replacement with plant matter on a mass basis would lead to large increases in calorie supply, with negative consequences for a population already facing a significant overweight and obesity burden (Ministry of Health 2020b), but this was not observed. This was due to the relatively low energy content of the selected plant foods; had a cereal-based scenario been chosen, energy supply would have increased more dramatically.

The scope of the alternative proteins market extends beyond meat substitutes. We considered also simulating replacement of dairy, but did not for the following reasons. Firstly, the replacement on a mass basis is not as straightforward as for meat; for example, it has been estimated that soy beverages contain around 9% soy material, rice around 20%, almond around 4%, etc. and that considerable variation exists between products available in NZ (Smith et al. 2022). Thus, any substitution simulation would have to appropriately scale the mass of the substitute product, with considerable uncertainty around these values. Secondly, dairy alternatives are most commonly produced from purified components of the source material, leading to the production of waste streams and changes in nutrient composition. These waste streams may or may not be consumed in other products, with high uncertainty around formats and location of consumption (particularly for products manufactured overseas). This leads to further uncertainty around the nutritional contribution of the source material. This combined uncertainty makes simulating dairy replacement scenarios less feasible. Dairy is also treated separately to other proteinaceous foods (e.g. meat, eggs, legumes) in the NZ Eating and Activity Guidelines, so should be treated separately in simulation studies (Ministry of Health 2020a). For other alternative proteins and substitution scenarios (such as egg substitutes, cell-based meat, etc.), there is either a lack of data on nutritional composition or limited impact expected due to the relatively low current nutritional contribution of the food to be substituted.

The aspect of processing must also be considered for these meat scenarios. Here, we have assumed that meat was replaced with plant foods with no nutritional content lost in processing. While this has been modelled as the healthiest manner of replacing meat with plant foods (Reynolds et al. 2023), it is not necessarily the most common. Highly processed meat alternatives will have different nutritional profiles (due to nutrient loss in processing and potential gain from fortification), leading to different scenario conclusions. Our aim was to simulate the total food nutrient availability of increasing supply of the plant foods, to then inform formulation or fortification strategies for consumer products.

Several groups have analysed the partial or complete replacement of meat in the NZ diet, largely from an environmental or population health perspective (Drew et al. 2020; Barnsley et al. 2021; McDowell et al. 2022; Reynolds et al. 2023). Environmentally, these studies have universally found reductions in greenhouse gas emissions in replacement scenarios (the environmental outcome consistently analysed), and gains for population health (the extent dependent on the replacement). Internationally, dietary optimisation studies have obtained similar results (Wilson et al. 2019; Perignon and Darmon 2022). While these studies usually ensure the nutrient adequacy of their scenarios, they take a dietary approach, rather than the national supply approach taken here. This distinction is important because dietary data uses food items at the point of consumption, thus with the benefit of any fortification built into the underlying data. Contrastingly, the supply approach shows nutrient availability in the absence of fortification, allowing identification of undersupplied nutrients that can then be addressed by product-level fortification or formulation. This is an essential complementary viewpoint, particularly for food industry and policy, when planning for the sustainability of future food systems, and the major contribution of this paper.

There is existing technology to fortify alternative proteins with the key nutrients identified here and its use is present in diverse consumer offerings on supermarket shelves in NZ (e.g. breakfast cereals fortified with iron and B vitamins, plant-based beverages fortified with calcium). Barriers to the use of fortification include negative consumer perceptions of the technology (Ipsos-Eureka 2010), impacts on the sensory properties of food (particularly for iron) (Hurrell 2022), and, from a manufacturer’s perspective, a lack of regulations and monitoring, insufficient public-private partnership, and little economic incentive (Olson et al. 2021). For NZ, these challenges can be exemplified by the difficulty in instituting the mandatory fortification of flour with folic acid. Despite approval and recommendation of this approach from the government agency responsible in 2007 and widespread mandatory fortification overseas, NZ did not adopt mandatory fortification until 2021, largely attributed to industry resistance (Thurston et al. 2023). This bodes poorly for future mandatory fortification policy, but should not deter government from progressing with evidence-based fortification policy where a strong public health case exists. It should also not prevent the strong encouragement of voluntary fortification programs.

Currently, NZ mandates fortification of wheat flour with folic acid and the use of iodised salt in breadmaking, and permits voluntary fortification of many products with various vitamins and minerals (e.g. breakfast cereals with B vitamins), and fluoride in bottled water. Comprehensive data on the extent of voluntary fortification is lacking, but an analysis of NZ packaged meat analogues found that only 17% of products were fortified with iron, vitamin B12, and zinc, all from the same manufacturer (Young et al. 2023). An analysis of plant-based beverages on the NZ market found that most were fortified with calcium, while only certain brands were further fortified with specific B vitamins (Smith et al. 2022). The limited extent of current fortification demonstrates the opportunity for increasing nutrient intakes via encouragement of further voluntary fortification.

The cost of fortification approaches is negligible, particularly in comparison to the calculated cost savings of adequate nutrition (Horton 2006; Olson et al. 2021). For example, Horton (2006) highlights the 70:1 benefit to cost ratio of salt iodisation in developing countries, and a 36:1 ratio for iron fortification, due to reduced healthcare costs and greater national productivity. This was at a cost of <1 USD per person per year. While these benefit to cost ratios may be lower in high-income countries like NZ, micronutrient deficiencies are still highly prevalent in these countries (Ministry of Health 2020b; Stevens et al. 2022), so the cost-effectiveness of fortification will remain true in these settings.

The scenarios analysed here identified the current state of nutrient supply in NZ, and how this might change under hypothetical future scenarios with reduced meat supply and increases in the supply of plant-based alternatives. The results emphasise the need for alternative protein producers to consider the micronutrient content of their products, and how this might be improved to meet existing and emerging nutrient insufficiencies. In particular, iron and zinc fortification should be prioritised in products designed as meat substitutes, while other nutrients (calcium, potassium, vitamins C and E) could be added to contribute to addressing existing undersupplies and dietary shortfalls.
